# Wound Healing Promotion by Hyaluronic Acid: Effect of Molecular Weight on Gene Expression and In Vivo Wound Closure

**DOI:** 10.3390/ph14040301

**Published:** 2021-03-28

**Authors:** Yayoi Kawano, Viorica Patrulea, Emmanuelle Sublet, Gerrit Borchard, Takuya Iyoda, Rihoko Kageyama, Asa Morita, Satoshi Seino, Hideto Yoshida, Olivier Jordan, Takehisa Hanawa

**Affiliations:** 1 Laboratory of Pre-Formulation Study, Faculty of Pharmaceutical Sciences, Tokyo University of Science, Chiba 278-8510, Japan; y.kawano@rs.tus.ac.jp (Y.K.); 3b12022@alumni.tus.ac.jp (R.K.); 2 Institute of Pharmaceutical Sciences of Western Switzerland, University of Geneva, 1 Rue Michel Servet, 1211 Geneva, Switzerland; viorica.patrulea@unige.ch (V.P.); Emmanuelle.Sublet@unige.ch (E.S.); gerrit.borchard@unige.ch (G.B.); 3 Section of Pharmaceutical Sciences, University of Geneva, 1 Rue Michel Servet, 1211 Geneva, Switzerland; 4 Faculty of Pharmaceutical Sciences, Sanyo-Onoda City University, Yamaguchi 756-0884, Japan; iyoda@rs.socu.ac.jp; 5 Kewpie Corporation, Tokyo 182-0002, Japan; asa_morita@kewpie.co.jp (A.M.); satoshi_seino@kewpie.co.jp (S.S.); hideto_yoshida@kewpie.co.jp (H.Y.)

**Keywords:** hyaluronic acid, accelerated wound healing, epidermal cells, cytokines

## Abstract

Hyaluronic acid (HA) has been known to play an important role in wound healing process. However, the effect of molecular weight (MW) of exogenously administered HA on the wound healing process has not been fully understood. In this study, we investigated HA with different MWs on wound healing process using human epidermal keratinocytes and dermal fibroblasts. Cell proliferation and migration ability were assessed by water soluble tetrazolium (WST) assay and wound scratch assay. We examined the effect of HA addition in a full-thickness wound model in mice and the gene expression related to wound healing. Proliferation and migration of HaCaT cells increased with the increase of MW and concentration of HA. Interleukin (IL-1β), IL-8 and vascular endothelial growth factor (VEGF) as well as matrix metalloproteinase (MMP)-9 and MMP-13 were significantly upregulated by high molecular weight (HMW) HA in keratinocytes. Together with VEGF upregulation and the observed promotion of HaCaT migration, HA with the MW of 2290 kDa may hold potential to improve re-epithelialization, a critical obstacle to heal chronic wounds.

## 1. Introduction

Skin wound healing is a dynamic and highly orchestrated process of cellular and molecular mechanisms that begins directly after an injury has occurred and might last for years depending on the type and size of the wound [1]. Wound restoration is divided into several main phases, which overlap over time, namely hemostasis, inflammation, proliferation/migration and maturation or remodeling [2,3]. During these phases, several elements are important to achieve early closure of the wound and scarless healing. For instance, platelet aggregation, release of proinflammatory cytokines (e.g., interleukin (IL-1β), IL-6, IL-8 and tumor necrosis factor (TNF-α)), growth factors (e.g., platelet-derived growth factor (PDGF), transforming growth factor beta (TGF-β), transforming growth factor alpha (TGF-α), basic fibroblast growth factor (bFGF) and insulin-like growth factor-1 (IGF-1)), which are very important during the inflammation phase [4]. Pro-inflammatory cytokines and growth factors play an important role for recruitment and activation of both epithelial and fibroblast cells, which prepare the wound site for the next healing phase. The proliferation stage overlaps with the migration of both keratinocytes and fibroblasts for the restoration of the vascular network and formation of granulation tissue. The restoration process of the vascular network or the formation of new blood vessels is also known as “angiogenesis” and is initiated by vascular endothelial growth factor (VEGF), PDGF, FGF and TGF-α [5]. The final step, maturation of the granulation tissue, involves the formation of the immature scar until the new tissue regains its integrity [6].

Hyaluronic acid (HA) is a natural and linear polysaccharide, consisting of repetitive disaccharide units of D-glucuronic acid and N-acetyl-D-glucosamine bound by β(1→3) and β(1→4) glycosidic bonds. Its molecular weight can range up to 10^8^ Da depending on the synthesis pathway [7]. HA is a major constituent of the extracellular matrix (ECM) in the human body; it is constantly synthesized as high molecular weight (HMW) HA and is degraded very fast by hyaluronidases [8]. Moreover, it plays an important role in supporting cells during the wound healing process [9,10], recognition by specific surface receptors during healing process [11], collagen deposition and angiogenesis [8]. HA is known to activate keratinocytes and is involved during proliferation, migration and tissue maturation phases of the healing process [12]. However, HA is rapidly metabolized in skin, its half-life is less than a day. HA is also actively degraded within 24 h by the hyaluronidase enzymes or by reactive oxygen species [7,12].

It has been confirmed that HA is involved in different stages of wound healing according to their individual roles. HA was also reported to promote healing of the fetal mouse limbs by inducing scarless repair [13]. High molecular weight (HMW) HA at the inflammation stage is aggressively decomposed into oligomers of low molecular weight (LMW) HA, which in turn promotes leukocyte chemotaxis and expression of inflammatory cytokines like IL-1β, TNF-α and IGF-1 [8]. HMW HA displays anti-angiogenic and anti-inflammatory properties, whereas LMW HA (<1000 kDa) acts oppositely, being pro-inflammatory and pro-angiogenic [8]. LMW HA by-products of HA degradation have key impact on healing, driving angiogenesis in the wound [14]. HA oligosaccharides trigger angiogenesis through endothelial cell proliferation through binding to HA receptors, such as CD44 or RHAMM [4,15].

The purpose of this research is to investigate and provide a full overview of the role and the influence of HA with different molecular weights (MWs) on wound healing in both in vitro and in vivo studies on mice. Although, there are many studies on HA in vivo, yet there is no published work to show the full picture on the influence of MW of HA on wound healing. Herein, in vitro assays on keratinocytes and fibroblasts are performed on a full range of MW of HA and concentration and the expression of nine key genes for wound healing (IL-1β, IL-6, IL-8, VEGF, MMP-2, MMP-9, MMP-13, TGF-β1 and TGF-3β) was evaluated using real-time PCR. In vivo experiments were further performed to validate the findings.

## 2. Results

### 2.1. Physicochemical Analysis of HA Ointments

Prior to administration, HA ointments were prepared and investigated for their physicochemical properties. Information on the spreadability, yield value, adhesion energy of the ointments with/without HA are reported in Table 1.

Throughout the data in Table 1, there were no significant differences between data for each measurement item, it is considered that the addition of HA does not affect the physicochemical properties of ointment.

As for the uniformity of HA in ointments, we confirmed by both naked-eye observation and the content uniformity of HA. Content rates of HA in each sample were 97.5–99.6%, it was considered that HA is uniformly dispersed in the ointment. Adhesiveness of the formulation increases with the increase of MW of HA, as expected. Moreover, spreadability values are similar to PBS, used as control, which indicates good spreadability. Still, the HA-K is the least spreadable due to its higher MW. Increase of HA bioadhesion with MW has been established in the field of ocular formulations [16] or more fundamentally in polymer science [17]. It is related to the density of mechanical tissue-polymer interlocking and availability of HA acidic moieties, in turn related to gel viscosity and HA molecular weight. Still, HA is a relatively low-adhesive biopolymer. Accordingly, in our previous study, when HA solution was applied onto bladder mucosa, the adhesion energy increased with HA concentration, which is expected since the density of HA molecules on mucosal surface affects the adhesiveness. From this point of view, the higher the molecular weight of HA, the more HA’s density on the surface.

### 2.2. In Vitro Release Profile of HA from the Ointment Formulation

**Figure 1:**
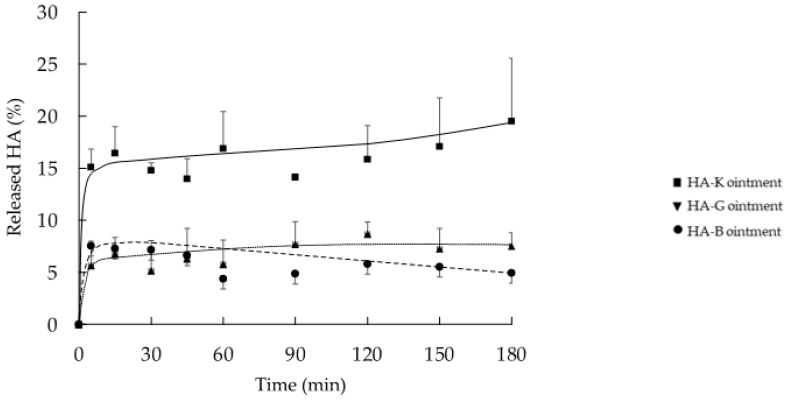
In vitro cumulated release fraction of HA from the various ointment formula HA-B (8 kDa), -G (987 kDa) and -K (2290 kDa) (*n* = 3, mean ± SEM).

Figure 1 shows the in vitro release behavior of HA from the various HA-containing ointments. In general, a fast release of HA with any MW from ointment is observed during the first 5 min: 5.7, 7.6 and 15.1%, corresponding to HA-B, -G and -K and afterwards followed by a plateau. However, the amount of HA released from the ointment containing HA-K (19.5%) tended to be higher than from HA-B (5.7%) or HA-G (7.6%). In a similar study (data not shown), we could observe a similar albeit slower release profile of HA from a cellulose sponge over 1500 min, which reached a plateau after 180 min. In this study, we applied a relatively thick layer of HA (1.5 g of ointment, 1.0 cm thickness) on the Franz diffusion cell for quantification purposes. The formulation already released 20% of its contents within a short period of time (3 h). In a clinical setup (thinner layer, >24 h application period) higher amounts of released HA are expected. Specific in vitro/in vivo release correlations would deserve further studies.

### 2.3. In Vitro Proliferation of HaCaT and NHDF Cell

As mentioned before, it is well known that at early stages of wound healing, the proliferation and migration of keratinocytes and fibroblast will help the regeneration of granulation tissue. At first, we investigated the possibility that HA promotes the proliferation of keratinocytes and/or fibroblast cells.

**Figure 2:**
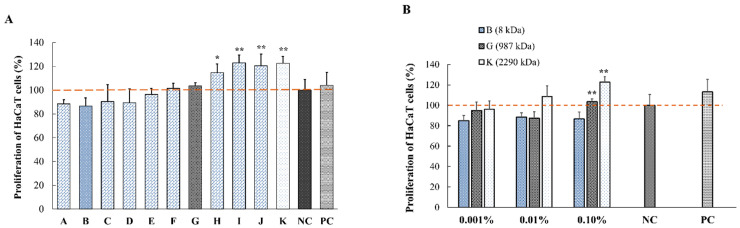
In vitro effect of HA with different MWs on the proliferation of HaCaT cells. (**A**) Incubation of HaCaT cells with 0.1% solutions of HA with different MW (HA-A:2, B:8, C:75 HA-D:300, E:619, F:800, G:987, H:1300, I:1530, J:1810 and K:2290 kDa) in complete DMEM for 48 h. Control groups include a negative control (NC group, 1% FBS-DMEM) and a positive control (PC group, 10% FBS-DMEM. The percentage of proliferation was calculated as the viability normalized to the NC group. (**B**) Incubation of HaCaT cells with 0.001%; 0.01% and 0.1% HA-B, -G and -K solutions (diluted in 1% FBS-DMEM) for 48 h (* *p* < 0.05, ** *p* < 0.01 vs. NC group using Dunnet’s test (*n* = 5)).

At first, we screened the whole range of MW HAs. We chose three different representative molecular weights: low, medium and high MW (LMW, MMW and HMW, respectively). Only HMW HA showed significant promotion of proliferation in HaCaT cells. The “K” group represents the HMW HAs, not being significantly different from the other HMW HA (-H, -I, -J) in terms of proliferation. The results from Figure 2 demonstrate that the proliferation of the HaCaT cells depends on the MW of HA. Proliferation rate of HaCaT cells increased with the increase of HA MW. Compared to the NC group, proliferation rate increased from 86% to 122% when increasing the MW of HA (HA-I, -J and -K) (Figure 2A). Significant increase was observed for MW above 1000 kDa, therefore, one representative of each group (HA-B, -G and -K) was retained for our in vitro and in vivo studies; however, HA-B was kept as group reference.

Therefore, subsequent experiments were carried out focusing on 3 types of HAs, HA-B (8 kDa), -G (987 kDa) and -K (2290 kDa), according to their MWs. To ensure absence of endotoxin in these selected HA, the fractions B, G, K were diluted with sterile endotoxin-free water and tested using a gel-clot endotoxin assay. None of the materials shown detectable bacterial endotoxin above the level of 0.25 EU/mL.

Figure 2B shows the effect of increasing HA concentrations from 0.001 to 0.1% with different MWs HA-B, -G and -K (in 1% FBS-DMEM) on HaCaT cells for 48 h. The enhanced proliferation of keratinocytes was observed upon increase of HA concentration. As only 0.1% HA showed significant effect on HaCaT cells, this concentration was selected for further studies. Of note, this is the highest concentration allowing to run migration assays, i.e., 0.1% for all MW range.

**Figure 3:**
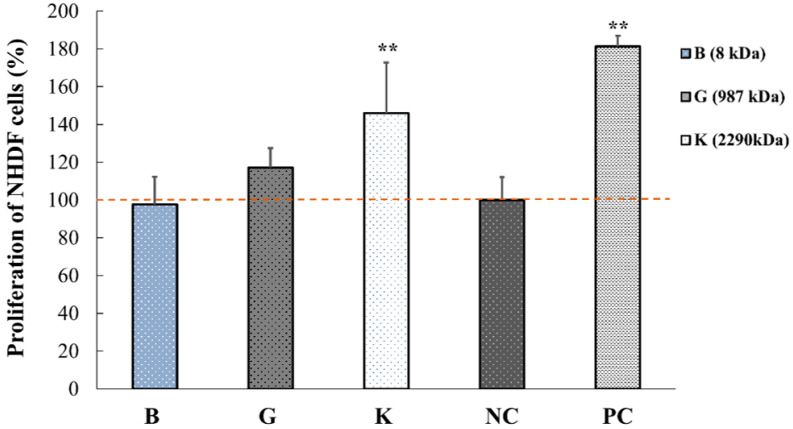
In vitro effect of three different MWs of HA on the proliferation of normal human dermal fibroblasts (NHDF). Control groups include negative control (NC group, 1% FBS-DMEM) and positive control (PC group, 10% FBS-DMEM). Data are expressed as percentage of number of cells (mean ± SEM) vs. NC group (*n* = 5, ** *p* < 0.01).

Figure 3 shows the effect of three different MW of 0.1% HA (HA-B, -G and -K) on the proliferation of NHDF for 48 h. Similar to keratinocytes, the proliferation of NHDFs was significantly increased (** *p* < 0.01 vs. NC) when increasing the MW of HA to 2290 kDa.

These results also suggest that the proliferation of HaCaT and NHDF cells depends on the MW of HA. More specifically, HA of 2290 kDa promoted the proliferation of both fibroblasts and keratinocytes. These data suggest that HA could contribute to the reduction of wounded area.

### 2.4. In Vitro Effect of HA on Keratinocytes or Fibroblasts Wound Closure

**Figure 4:**
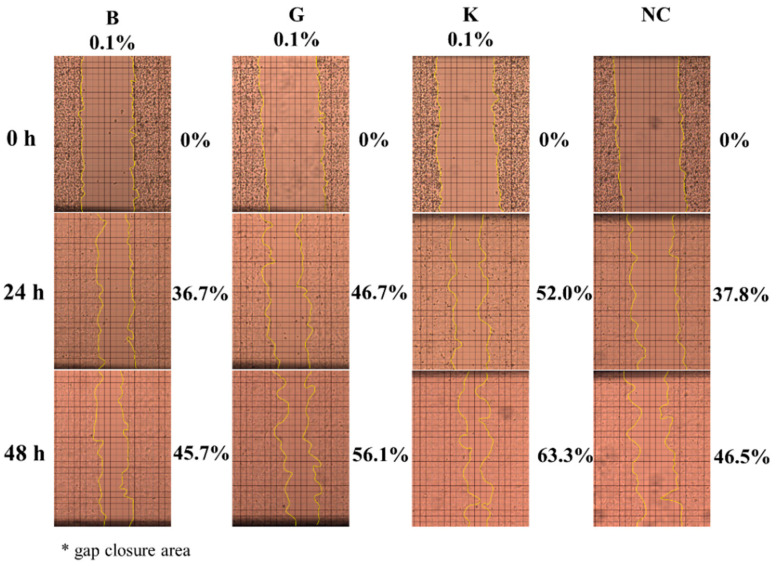
HaCaT cells migration investigated by in vitro wound scratch assay using HA-B (8 kDa), -G (987 kDa) and -K (2290 kDa) at different time points. Representative images of HaCaT cells monolayer with scratch after culturing for 0, 24 and 48-h with/without 0.1% HA at three different MWs.

Figure 4 shows the HaCaT cells migration investigated by in vitro wound scratch assay using HA-B, -G and -K at different time points. When comparing these three HAs of different MWs, the gap of the wound at 6, 24 and 48 h decreased with the increase of both MW and the concentration of HA.

**Figure 5:**
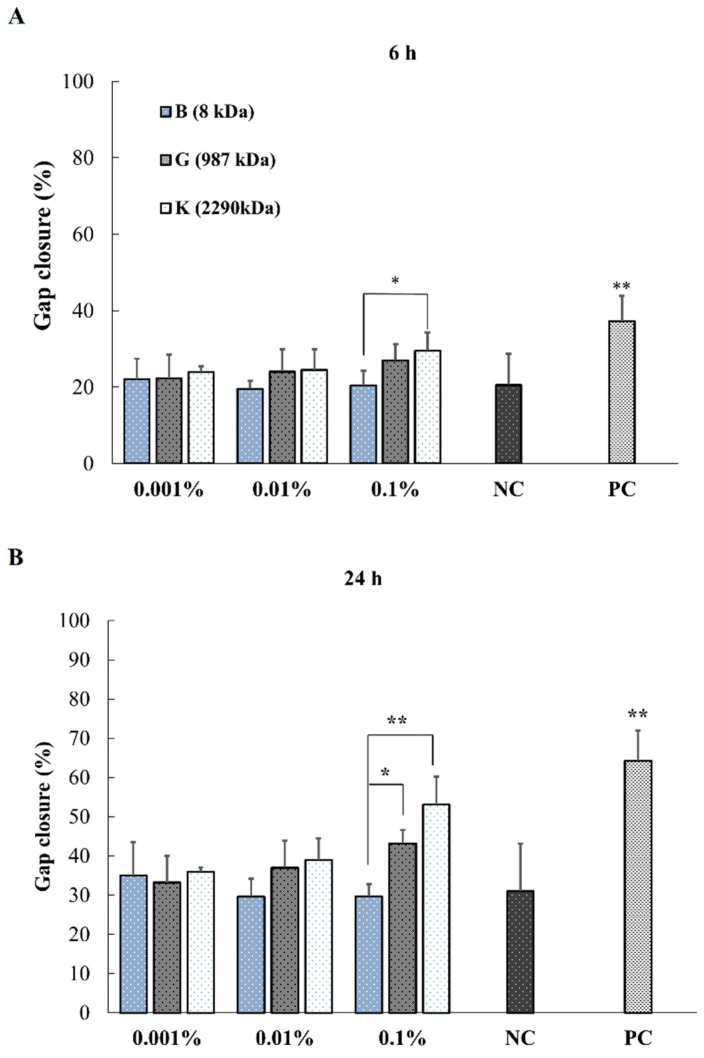

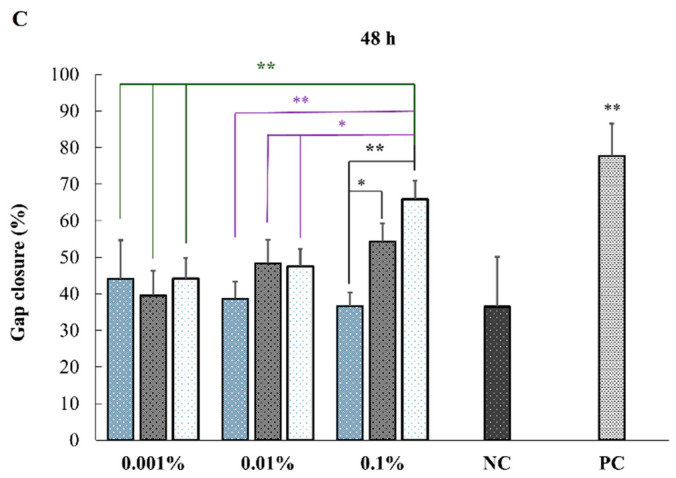
Representation of in vitro wound closure of HaCaT cells at time 6 h (**A**), 24 h (**B**) and 48 h (**C**) upon addition of 0.001, 0.01 and 0.1% HA with three MWs. Data are expressed as wound closure percentage of wound scratch area relative to initial scratch area (mean ± SEM, *n* = 5, * *p* < 0.05, ** *p* < 0.01).

More specifically, at 0.1% concentration, a significant effect of MW was observed for all time points excepted between -B and -K at 6 h (Figure 5A). HMW HA-K showed higher promotion ability for wound closure than MMW HA-G and LMW HA-B and NC at 24 and 48 h (*p* < 0.01 and 0.05) (Figure 5B,C) except for K vs. B at 6 h (Figure 5A).

**Figure 6:**
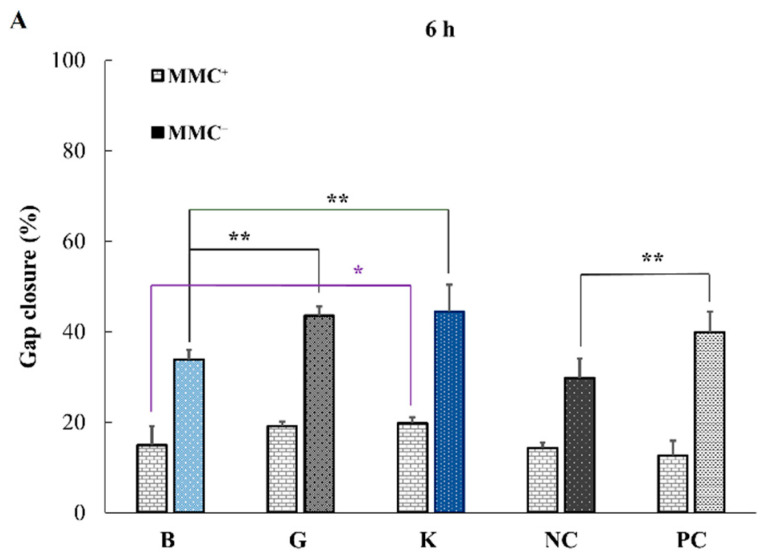

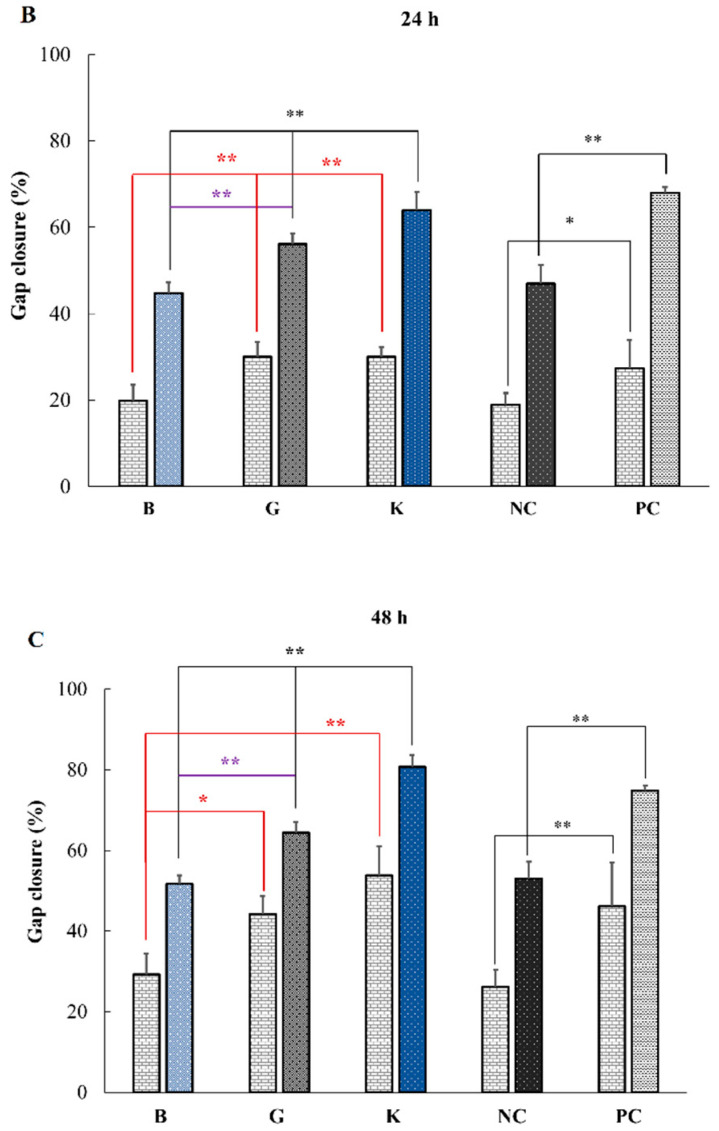
Effect of the addition of 0.1% HA on the migration of HaCaT cells towards gap closure with/without MMC in the wound scratch assay after 6 h (**A**), 24 h (**B**) and 48 h (**C**) using HA-B (8 kDa), -G (987 kDa) and -K (2290 kDa). Data are expressed as percentage of scratch wound healing area (mean ± SEM, *n* = 5, * *p* < 0.05, ** *p* < 0.01, Dunnet test).

In a wound scratch assay, because the wound healing relates not only to cell migration but also to their proliferation, mitomycin C (MMC) was added as an inhibitor for DNA replication [18]. Therefore, besides leaving the cells under serum starving conditions (1% FBS), MMC was additionally used to control the proliferation upon HA addition and investigate the migration alone. The experiments were performed in the presence (MMC+) or absence (MMC−) of 50 μg/mL of antimitotic MMC after 2 h incubation in DMEM supplemented with 1% FBS. The gap closure was monitored at time points 0, 6, 24 and 48 h of culturing with/without 0.1% HA of different MWs (Figure 6).

Figure 6 shows the effect of HA addition on the migration of HaCaT cells towards closure of the gap by wound scratch assay performed in the with/without of MMC (MMC+ or MMC-), respectively. The fact that the wound gap decreased with the increase in MW of HA in the presence of MMC indicates that cell migration is promoted by the addition of higher MW HA. Specifically, a significant (*p* < 0.01) increase in wound closure was observed between B and G and between B and K at 24 and 48 h, as well as between B and K at 6 h (Table 2).

**Figure 7:**
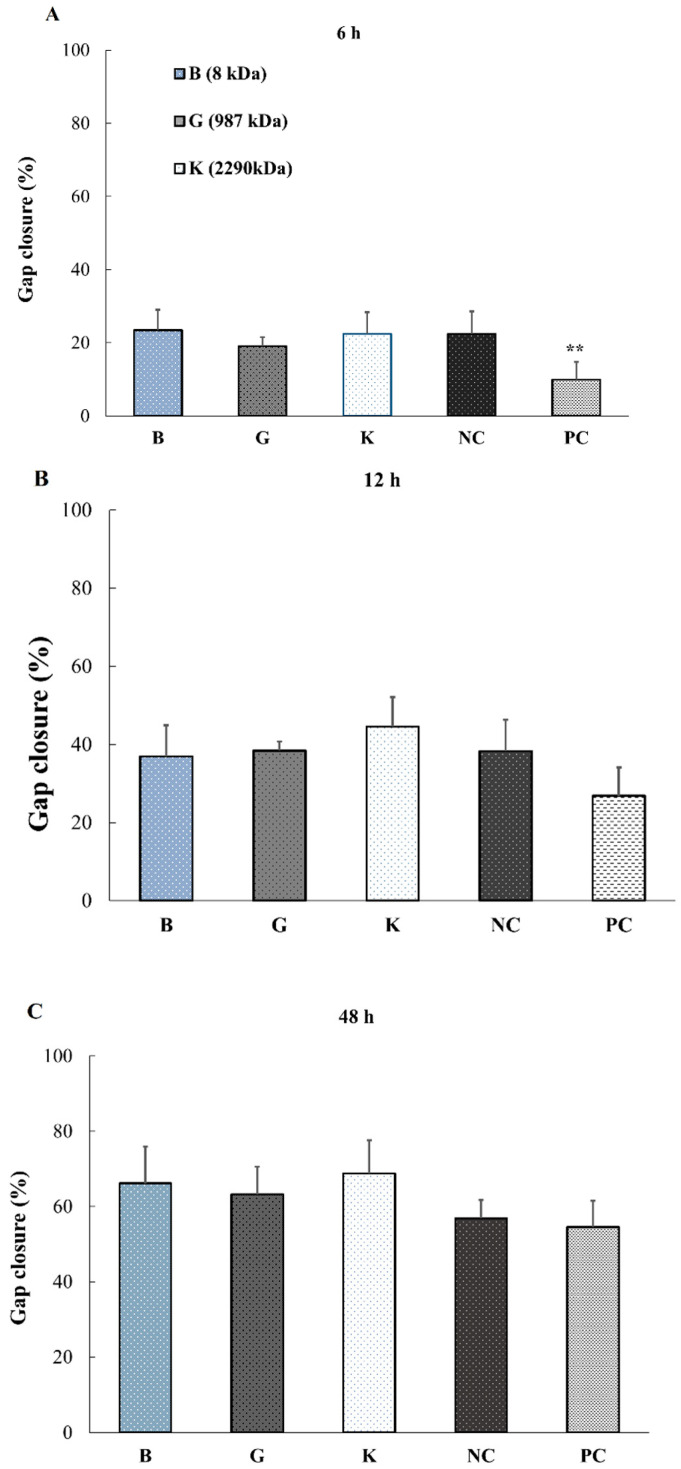
Fibroblast wound closure without MMC evaluated by wound scratch assay at different time points: 6 h (**A**), 12 h (**B**) and 48 h (**C**) using HA-B (8 kDa), -G (987 kDa) and -K (2290 kDa) (0.1% HA, *n* = 5, ** *p* < 0.01 vs. NC group, Dunnet test).

Successively, the migration and proliferation ability of the fibroblast cells (NHDF) without MMC was further evaluated. Surprisingly, NHDF behaved differently from keratinocytes. The wound closure did not depend on the MW of HA and no significant differences were observed for any of the HAs regardless of their MWs (Figure 7).

These results suggest that HMW HA (>987 kDa) promotes the migration of epidermal cells, which in turns leads to a decreased wound area. Especially since the promotion of cell migration by HMW HA was seen within 6 h, it suggests that HA-K could be beneficial for wound healing by promoting cell migration during an early stage of healing.

### 2.5. Effect of HA Addition on the Promotion of Wound Healing

In vitro WST-8 and scratch-wound assay revealed that increasing the MW of HA accelerates the reduction of wound size. In this study, in order to clarify the effect of the differences between different MW exogenously administered HA on the wound size reduction in vivo, we used a mouse full-thickness wound model and the various MWs of HA (HA-B, -G or -K) ointments were exogenously administered.

**Figure 8:**
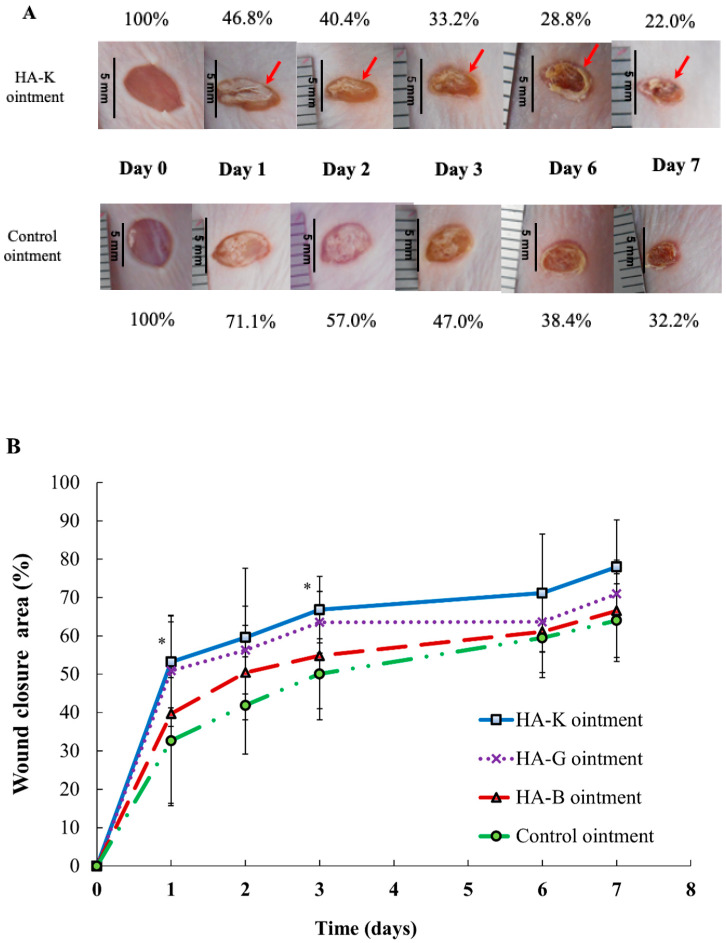
In vivo effects of HA ointments at 0.1% on healing of full-thickness excisional. The ointments have been applied daily. (**A**) Representative photographs for the full-thickness wounds in hairless mice at different time points. Macroscopic changes in skin wound sites induced by topical application of the control and HA-K ointment at day 0 (picture taken immediately after injury), 1, 2, 3 and 7. (**B**) Graphical representation of wound closure after topical application of control ointment (control group), HA-B, HA-G and HA-K ointment at day 0, 1, 2, 3, 6 and 7. Encrusted wound sites are represented with a red arrow. Data are expressed as percentage of wound area from the initial wound size (day 0). Values are shown as mean ± SEM (*n* = 4 wounds/group), * *p* < 0.05 vs. control group.

Figure 8A shows the representative photographs of the macroscopic changes at the skin wound site after topical application of Control or HA-K ointment at day 0 to 7. Ointments based on HA-B and -G did not show any wound size reduction at day 7 (Figure S2). Figure 8B shows the wound area reduction induced by topical administration of Control ointment, HA-B, -G or -K ointment. Especially, the reduction of wound size only in the HA-K treated mice was higher than in the control group. Wound closure was significantly higher (*p* < 0.05) compared to the control group at day 1 and 3. Furthermore, in case of HA-K administration, because the wound site was shrunk at day 1 already, we assume that the application of ointment lead to crust formation from day 2. Therefore, among all HA ointments applied in this study, the formation rates of the crust were higher in the group of mice treated with HA-K and -G at day 1, which indicates that wound healing started at an early stage after the application of HA ointment.

### 2.6. Gene Expression Analysis on Keratinocytes and Fibroblasts Exposed to HA

To obtain a deeper insight into how HAs promote the wound healing process, we next evaluated the expression of several genes that might be responsible candidates for the promotion of wound closure. Based on previous reports [19], nine genes listed in Table 3 and Table 4 were chosen as target and their mRNA expression were evaluated by real-time PCR analysis involving HaCaT and NHDF cell monolayers with/without multiple scratch.

**Figure 9:**
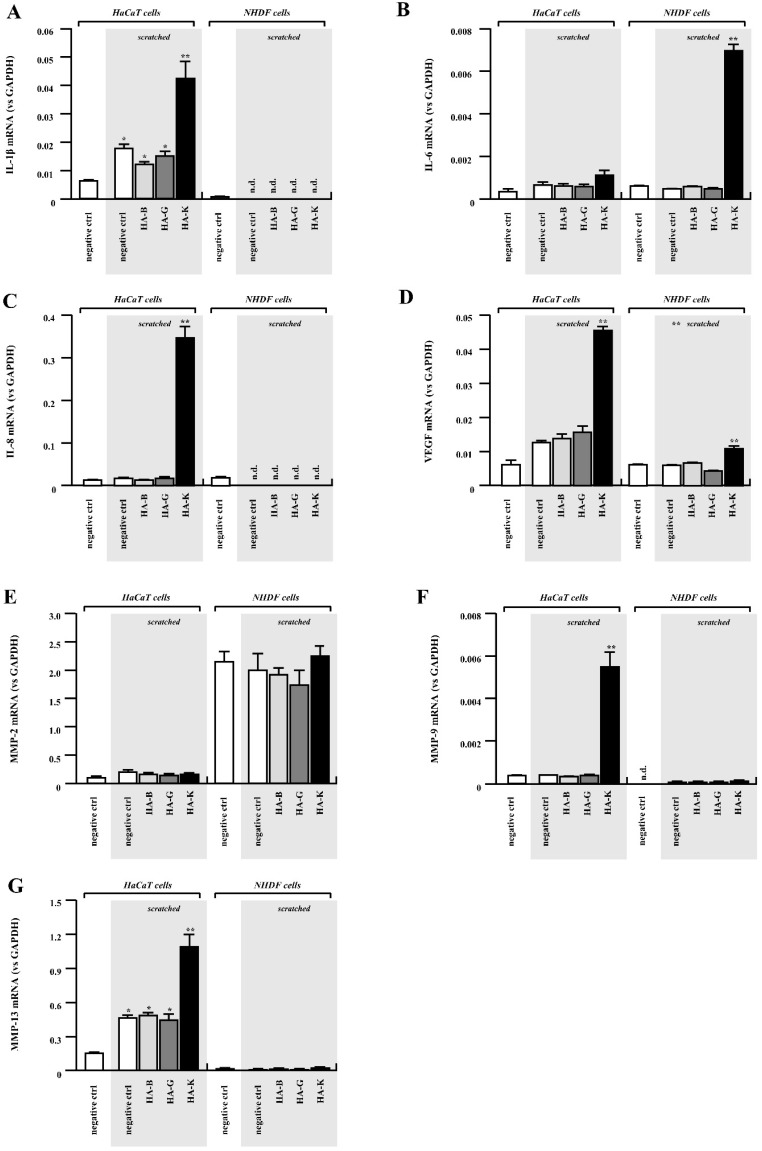

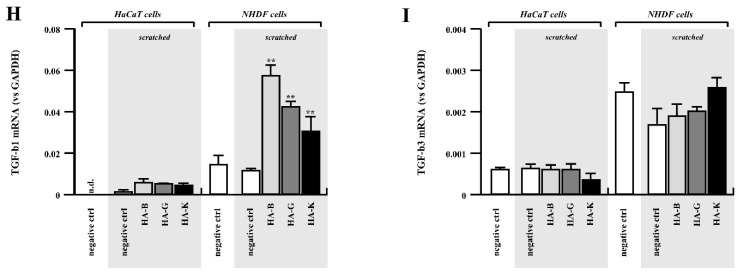
Gene expression induced by HA administration in HaCaT and NHDF cells. Gene expression of IL-1β (**A**), IL-6 (**B**), IL-8 (**C**), VEGF (**D**), MMP-2 (**E**), MMP-9 (**F**), MMP-13 (**G**), TGF-β1 (**H**) and TGF-3β (**I**). Results from HaCaT cells are shown on the left side and from NHDF cells are on the right side. Relative gene expression level (vs GAPDH) from scratched cells is shown on grey background. Relative mRNA expression of cells with 0% HA is filled in white bar, 0.1% HA-B is in light grey, 0.1% HA-G is in grey and 0.1% HA-K is in black bar. Data are expressed as mean ± SEM. * *p* < 0.05 vs. unscratched control group (** *p* < 0.05 vs. 0% HA control group, *n* = 5).

First, we focused on typical inflammatory cytokines IL-1β, IL-6 and IL-8. These molecules have been reported to contribute to wound healing by modulating not only leukocyte recruitment, but also keratinocyte and fibroblast proliferation and their migration. Among these genes, IL-1β and IL-8 were significantly upregulated by the addition of high MW HA-K when HaCaT cell monolayers were “injured” by multiple scratching (Figure 9A,C). On the other hand, expression of these two genes in NHDF cells was relatively lower than in HaCaT cells and were not affected by addition of HAs (Figure 9A,C). This may account for the specific promotion of migration observed for HaCaT cells, but not for NHDF.

In sharp contrast to IL-1β and IL-8 expression, IL-6 was significantly upregulated in NHDF cells treated with HA-K ointment (Figure 9B). HA-K mediated IL-6 upregulation in HaCaT cells was observed as well, even though the magnitude was much lower than in NHDF cells (Figure 9B). Significant upregulation of VEGF was observed only in HA-K treated HaCaT cells with multiple scratch (Figure 9D), similarly to another gene implied in neovascularization, IL-8. All the results are summarized in Table 3, which show the strong effect of HA-K on healing-related gene expression for HaCaT cells. One should take in consideration that there are many differences between foetal and adult cells in expressing cytokines [19].

ECM degradation is one of the key events rendering wound healing efficient through acceleration of ECM regeneration and cell migration. Moreover, matrix metalloproteinases (MMPs) play an important role during wound healing [19]. As shown in Figure 9F,G, MMP-9 (gelatinase-B) and MMP-13 (collagenase) were significantly upregulated by addition of HA-K to the keratinocytes. On the contrary, fibroblasts did not show significant changes in the expression of these two genes even when stimulated with HAs after mimicking the wound by scratch formation (Figure 9F,G).

We observed an upregulation of MMP-2 in NHDF, consistent with the known upregulation of elastase-type endopeptidases in human skin fibroblast upon HA exposure [20]. Moreover, MMP-2 is known to be highly expressed by fibroblasts during the inflammation phase, which is critical for further tissue maturation [21]. TGF-β1 promotes the proliferation, collagen formation and differentiation of dermal fibroblasts and can stimulate fibroblast migration by up regulation of MMPs such as MMP-2 [22]. Such activation of the TGF-β/MMP-2 signaling pathway, which promotes cell motility, may be attributed to HA/CD44 activation [23]. On the other hand, it is well-known that MMPs’ inhibition or absence would lead to progression to chronic inflammation [24].

Contribution of TGF-β to wound healing has generally been well accepted [25]. Under our experimental conditions, TGF-β1 was upregulated in NHDF cells after HA addition (HA-B, HA-G and HA-K, Figure 9H). HA-K mediated upregulation of TGF-β1 in HaCaT cells as well, although with a much lower magnitude than in NHDF cells (Figure 9H).

We also performed the analysis for MMP-2 (gelatinase-A) and TGF-β3, as one of MMP and TGF-β family members. However, there were no significant differences even when HaCaT cells were cultured with HA or cells were “injured” by scratch formation. Almost the same behavior was observed for NHDF cells, although basal expression levels of these two genes were relatively higher in NHDF cells than HaCaT cells (Figure 9E,I).

## 3. Discussion

It has been reported that high levels of macromolecular hyaluronic acid lead to decrease scarring, whilst the adult phenotype is characterized by increased numbers of breakdown products and smaller molecules [26]. In addition, it has been reported that inflammation is induced when HA fragments are broken down by hyaluronidase [27]. Based on these reports, HMW-HA added without hyaluronidase is considered to enhance epidermal cell proliferation. Our results obtained from the in vitro WST-8 assay and scratch-wound assay suggest that the proliferation and the cell migration ability were promoted by the increase in MW of HA in a dose-dependent manner without hyaluronidase. Furthermore, real-time PCR results proved that the HMW HA promotes not only the proliferation of HaCaT cells, but also the expression of different genes responsible for the formation of extracellular matrix, angiogenesis and leukocyte chemotaxis.

These observations suggest the possibility that HA-K promoted efficient wound healing as observed during this in vivo study and that wound healing may be mediated by upregulation of several genes responsible not only for proliferation and migration of keratinocyte, but also for ECM regeneration, leukocyte recruitment and neovascularization. At last, HA-K mediated keratinocyte proliferation and migration was promoted by the enhanced expression of the genes investigated in this study. Since IL-8 and IL-6 are known to be upregulated and be able to activate proliferation/migration of keratinocytes and fibroblasts, respectively, it seemed that IL-8 and IL-6 would work through autocrine mechanism during the HA-K accelerated wound healing. In addition, HaCaT cells demonstrated an overexpression of IL-8 and VEGF. IL-8 is known to promote neovascularization and cell chemotaxis [28]. Together with VEGF upregulation and the observed promotion of HaCaT migration, the HA-K may hold potential to improve re-epithelialization, a critical obstacle to heal chronic wounds.

From these results, it is assumed that HMW HA influences the signaling of epidermal and dermal cells. The most representative HA receptor is CD44, which triggers differentiation in human keratinocytes [29] and fibroblast cells [30,31]. CD44 exists on the surface of cell membrane and is the adhesion molecule for cell-cell or cell-ECM contacts. It is reported that HA shows a high binding affinity to CD44 in fibroblasts and resistance to dissociation as its molecular weight increases [32]. By binding to HA, CD44 promotes cell proliferation, induction of differentiation and cell migration and is involved in promoting the induction of inflammatory cytokines or MMPs [33]. HMW HA stimulates CD44 clustering, in contrast to LMW HA [30]. Since it is considered that exogenously administered HA binds to CD44 in epidermal or fibroblast cells [34], genetic expression relating to the acceleration of wound healing appears to be promoted by signal transduction of CD44.

## 4. Materials and Methods

HA of different molecular weight (MW, weight average), named HA-A, B, C, D, E, F, G, H, I, J and K (MW: 2; 8; 75; 300; 619; 800; 987; 1300; 1530; 1810 and 2290 kDa, respectively) were provided by Kewpie Corporation (Tokyo, Japan). The MWs of HA were determined based on Mark–Houwink–Sakurada relation using their intrinsic viscosity [35]. Fetal bovine serum (FBS) was purchased from GE Healthcare (Buckinghamshire, UK). Dulbecco’s modified Eagle’s medium (DMEM) and Mitomycin C were purchased from Wako Pure Chemical Corporation (Osaka, Japan). Dulbecco’s phosphate-buffered saline (PBS) was purchased from Funakoshi Co., Ltd. (Tokyo, Japan). Trypsin-ethylenediamine-*N*, *N*, *N’*, *N’*-tetraacetic acid (EDTA) was purchased from Thermo Fisher Scientific (MA, USA). Penicillin and streptomycin were purchased from Sigma-Aldrich (St. Louis, MO, USA). Cell Counting Kit was purchased from Dojindo Laboratories (Kumamoto, Japan).

Purified, i.e., allergen- and alcohol-free lanolin and Plastibase^®^ were purchased from Yoshida Pharmaceutical Co., Ltd. (Tokyo, Japan) and Taisho Pharmaceutical Co., Ltd. (Tokyo, Japan), respectively.

The Japanese Pharmacopoeia, 17th Edition (JP17) 2nd fluid for dissolution test and tetrahydrofuran were purchased from Kanto chemical Co., Inc. (Tokyo, Japan).

Hairless mice (male, HOS:HR-1) were purchased from SANKYO Labo Service Corp., Inc. (Tokyo, Japan).

Human-derived epidermal keratinocytes HaCaT cells (Tsukimoto laboratory (Department of Radiation Bioscience), Tokyo University of Science, Japan) and normal human dermal fibroblasts (adult donor NHDF, Takara Bio Inc., Shiga, Japan) were cultured at 37 °C and 5% CO_2_ in DMEM supplemented with 10% fetal bovine serum (FBS) and 1% penicillin-streptomycin.

### 4.1. Physicochemical Analysis: Assessment of Adhesiveness and Spreadability and Content Uniformity of HA Ointments

In this study, purified lanolin was used as emulsifying agent for W/O biphasic, which has ability to absorb water and is therefore suitable for mixing into an oily ointment base (Plastibase^®^), i.e., HA solution was absorbed by the purified lanolin, then mixed into Plastibase^®^. Specifically, HA with various MWs (6 mL) solutions were absorbed into purified lanolin (3.33 g) and mixed using a pestle and mortar. Then, Plastibase^®^ (20.67 g) was added and mixed. The final HA concentration in the ointment was adjusted as 0.1%. Control ointment was prepared using similar protocol containing 6 mL of PBS instead of HA.

The physicochemical properties of each HA ointment for in vivo study were evaluated by adhesiveness, spreadability and content uniformity.

The measurements of adhesive force were performed with using a creep meter (Yamaden, model 33005S, Tokyo, Japan) at room temperature (22 °C) [36]. The apparatus and procedures are schematically illustrated in Figure S1. A fixed volume (20 mL) of the HA ointment was weighed in a stainless Petri dish (45 mm diameter, 25 mm depth). In these assessments, we designated a Teflon^®^ plunger (20 mm diameter) that was lowered onto the surface of the HA ointment. The top of the plunger was dipped to a depth of 2 mm, the plunger was pulled up at a constant displacement rate of 1 mm/s. The adhesive force and the displacement were measured when the plunger was completely separated from the surface of the HA ointment. At the curve under the X-axis, the value of the load represents the tension received by the plunger and the peak area under the curve indicates the adhesion energy between the surface of the plunger and that of the samples; the larger the area of the load–strain curve, the higher the adhesion energy of the sample (Figure S1).

Spreadability was measured at 25 °C by a Spread Meter (Rigo Co., Tokyo, Japan). A definite volume (0.5 cm^3^) of sample was filled into the cylindrical hole and 115 g of glass plate was set just above the hole. The sample was pushed up and at the same time, glass plate was dropped at a distance of 5 cm on the surface of sample to have to be pinched spread the sample. The spread diameter was measured after 10, 20, 30, 40, 50, 60, 90, 120, 150, 180, 240 and 300 s. Yield values were computed from the following formula using the value at 300 s.

$$F=47,040\times G\;\times \frac{V}{\pi^{2}}\times D^{5}$$

where *F*: yield value (dyne/cm^2^), *G*: weight of the glass board (g), *V*: amount of sample (cm^3^) and *D*: the diameter when a spread of a sample stops (cm).

Content uniformity of HA in ointment was determined for each ointment. A definite weight (300 mg) of ointment was dissolved in 10 mL of tetrahydrofuran.

Throughout this study, HA concentration in various samples was determined by the Carbazole-sulfate method [36]. Briefly, 0.3 mL of sample solution and 3.0 mL of sulfuric acid solution (distilled water: H_2_SO_4_ = 1:8 (vol.:vol.)) were mixed and heated in a hot water bath for 10 min. Then, the solution was cooled in a water-ice bath and 0.3 mL of carbazole methanol solution (5 mg/mL) was added, followed by heating in a hot water bath for 15 min and cooling in a water-ice bath. After cooling, the absorbance of the absorbance was measured at 530 nm using an ultraviolet-visible spectrophotometer (UV-1800, Shimadzu Corporation, Japan).

### 4.2. In Vitro Release Profile of HA from Ointment Formulations

Release behavior of HA-B, -G or -K from HA ointments were investigated using the vertical type Franz-type diffusion cell. The 0.1% HA ointment was applied via the donor compartment, which is separated from the receptor chamber (filled with JP17 2nd fluid for dissolution test) by a polyethylene membrane (*ϕ* = 0.8 μm) The experiments were carried out in triplicates, at 32 °C and under continuous stirring using a magnetic stirrer. Aliquots of 0.3 mL were withdrawn at 5, 15, 30, 60, 90, 120, 150 and 180 min and replaced with fresh PBS. The sample collection was followed after supplementation with the same volume of PBS.

### 4.3. Endotoxin Assay

Bacterial endotoxin levels were assayed according to the manufacturer’s instructions for ToxinSensor Gel Clot Limulus Amebocyte Lysate (LAL) Endotoxin assay kit (Genscript Biotech, Netherlands; Cat. No. L00351) with a sensitivity of 0.25 EU/mL. Both positive (E. coli endotoxin standard at 0.5 EU/mL) and negative controls (LAL water) were included. HA samples were prepared in sterile endotoxin-free water at highest concentrations of 0.1% and incubated for 60 min at 37 °C.

### 4.4. Effects of HA Addition on Cell Proliferation In Vitro

#### 4.4.1. In Vitro Cell Proliferation Assay (WST-8 Assay)

HaCaT and NHDF cell viability and proliferation was assessed using WST-8 assay. Cells were seeded at a density of 5 × 10^4^ cells/mL in a 96-well plate and incubated for 12 h (5% CO_2_, 37 °C). After incubation, the medium was replaced by 100 μL of different HA solutions (0.001, 0.01 and 0.1% in DMEM with FBS 1%) and incubated for 48 h. WST-8 reagent was added to the cells and the absorbance of the samples was measured with an Infinite® 200 PRO spectrophotometer (Tecan Group Ltd., Männedorf, Switzerland) at a wavelength of 450 nm. Control groups in all in vitro assays include a negative control (NC group, 1% FBS-DMEM) and a positive control (PC group, 10% FBS-DMEM). Additionally, it was checked whether the viability of cells exposed to SDS 1% was lower than 5%.

#### 4.4.2. In Vitro Wound Scratch Assay

HaCaT or NHDF cells were seeded in a 24-well plate at a cell density of 3 × 10^5^ cells/mL until completely confluent cell monolayer was obtained. In order to distinguish between proliferation and migration phases, 50 µL/mL of antimitotic mitomycin C (in DMEM) was used (reaction time: 2 h) as inhibitor for DNA replication [18]. The cell monolayer was scratched in a straight line with a p1000 micropipette tip. HA solutions (0.001, 0.01 and 0.1%) were added on top of the “wounded” cells and the gap closure was followed at time points 0, 6, 24 and 48 h using bright-field microscopy (Nikon Eclipse Ts2, Nikon Corp., Tokyo, Japan). NC represents the “negative control” group, which are cells treated DMEM-1% FBS. Gap closure data are expressed in percentage of the area relative to the initial scratch area and compared to the NC group.

#### 4.4.3. Realtime-PCR Analysis

HaCaT or NHDF cells were seeded in a 6-well plate and cultured in complete medium. After forming a confluent cell monolayer, multiple scratch wounds (five lines for both horizontal and vertical direction) were created using p200 pipette tip. Cells were then washed twice with serum-free medium to remove cell debris followed by adding assay medium containing 0.1% HA or 1% FBS. After 4 h of incubation, cells were washed twice with PBS and subjected to RNA purification. Total RNA was extracted using GenElute Total RNA Purification Kit (Sigma) and cDNA was synthesized from approximately 0.5 µg of purified total RNA using QuatiTect Reverse Transcription Kit (Qiagen). Then, the expression of target genes was analyzed by CFX connect Real-time PCR system (BIO-RAD) using Thunderbird SYBR qPCR Mix (Toyobo). The expression levels of cytokines mRNA were normalized using mRNA of glyceraldehyde-3-phosphate dehydrogenase (GAPDH). Primer sequences are presented in Table 4.

### 4.5. In Vivo Wound Healing Experiments in Mice

Hairless mice (male, HOS:HR-1, medium average of weight: 27 g) were used for the in vivo experiments (Protocol Y16005 approved by Ethical Committee of Tokyo University of Science, Japan). Use of hairless mice avoid the impact of hair follicles cycle phase on wound healing. During the protocol, animals were housed in a 12-h automatic light-dark cycle (temperature 24 ± 1 °C and relative humidity 55 ± 5%). Food and water were given ad libitum.

Full-thickness wounds of 6 × 6 mm^2^ were created on the back of the anesthetized mice after aseptically cleaning with alcohol. Wounds were created with a biopsy punch (Kai Industries Co. Ltd., Gifu, Japan) on the right and left side (25 mm from the tail base, 10 mm from the spine) of each animal. A total of 0.2 mL of ointment containing HA-B, -G or -K or the control ointment were applied daily on the wounded area until Day 7. The major and minor axis were measured with a caliper at defined time points (Day 0, 1, 2, 3, 6 and 7). The injured area at day 0 was defined as 100% and the wound closure (%) was calculated following the equation:

$$Wound\;closure\;\left(\%\right)=\;1-\frac{Wound\;area\;at\;Day\;X\;\left(mm^{2}\right)}{Wound\;area\;at\;Day\;0\;\left(mm^{2}\right)}\;\times 100.$$

### 4.6. Statistical Analysis

All data were expressed as the means ± SEM (standard error of the mean). Two-way ANOVA analysis was performed with Tukey–Kramer and Dunnet’s post hoc test. The Dunnett’s multiple comparison test was used to assess differences. Student’s *t*-test was used in in vivo study that was compared versus the control. A value of *p* < 0.05 was considered statistically significant. Experiments were performed with *n* = 3–5 replicates.

## 5. Conclusions

HA, a key component of ECM, plays different roles in wound healing, i.e., promoting the expression of inflammatory cytokines such as IL-1β and TNF-α, triggering angiogenesis and activating keratinocytes and fibroblasts during healing process to promote wound healing. HA was known for decades for its remarkable properties in wound healing, but the full picture of screening the ability HA over all MW range, starting with the ultra-low until HMW is missing.

In this study, we investigated the effect of the exogenously administered HA on the factors associated with wound healing in vitro and in vivo with full-spectrum of MW and various concentrations of HA. In vitro WST-8 assay showed that HA, especially HMW HA promoted fibroblast and keratinocyte proliferation, a very important feature for the formation of granulation tissue. Moreover, cell proliferation was accelerated at MW > 987 kDa and strongly correlated to MW of HA.

In vitro assays showed that HMW HA was the most potent candidate to enhance keratinocytes migration, followed by MMW. Surprisingly, fibroblasts did not show any dependency on the MW of HA.

Based on screened genes, the results showed that IL-1β, IL-8 and VEGF as well as MMP-9 and MMP-13 were significantly upregulated by HA HMW in keratinocytes, suggesting HMW HA benefits for the treatment of wounds. NHDF did not show significant gene expression enhancement except for TGF-β1 which was upregulated preferentially by LMW HA.

Our results showed as well that exogenously administered HMW HA was highly effective for the treatment of wounds on mice. In the progress of wound healing, we suggest that exogenously administered HA promotes wound healing through interaction with CD44 expressed on keratinocytes and fibroblasts. Future studies might investigate whether the results obtained from these in vivo and in vitro studies and from real-time PCR are related to the CD44-HA interactions.

## Figures and Tables

**Table 1:** Physicochemical properties of HA ointments (mean ± SEM, *n* = 3, ANOVA, Tukey-Kramer post hoc test, no significant difference at *p* < 0.05).

Sample	Spreadability (mm^2^)	Yield Value (dyne/cm^2^)	Adhesion Energy (J/m^3^)
PBS	760.9 ± 0.06	925.0 ± 21.33	1960 ± 99.25
HA-B	780.3 ± 0.40	921.8 ± 52.02	1920 ± 271.8
HA-G	752.7 ± 0.56	881.4 ± 27.46	2030 ± 65.07
HA-K	791.4 ± 0.01	850.2 ± 7.35	2120 ± 58.31

**Table 2:** Significant level for HaCaT migration assay, in presence of mytomycin (*n* = 5, ANOVA, Dunnett multiple comparison test with *p* < 0.05 = *, *p* < 0.01 = **).

MMC+ Results	6 h	24 h	48 h
B vs. G	ns	**	*
B vs. K	*	**	**
G vs. K	ns	ns	ns

**Table 3:** Summary of the effect of HA-B, -G and -K on gene expression of HaCaT cells responsible for wound healing.

Genes Influencing Wound Healing	HA		
	B	G	K
**TGF-β1**	↑↑	↑	↑
TGF-β3	=	=	↓
**VEGF**	=	↑	↑
**IL-1β**	↓	=	↑
IL-6	=	=	↑
**IL-8**	↓	=	↑↑↑
MMP-2	=	=	=
**MMP-9**	=	=	↑↑↑
**MMP-13**	=	=	↑

“↓” stands for a decrease by 0.6–0.8 fold vs. control; “=” for 0.8–1.2 fold vs. control; “↑” for an increase by 1.2–5 fold vs. control; “↑↑” for an increase by 5–10 fold vs. control and “↑↑↑” is for >10 folds vs. control. HA-K results are highlighted in blue, suggesting the strong effect of HA on wound healing. The most important cytokines for wound healing are highlighted in red.

**Table 4:** Primer sequences used for realtime-PCR analysis.

Genes	Forward Primer	Reverse Primer
[Target]		
TGF-β1	GCCCTGGACACCAACTATTGC	GCACTTGCAGGAGCGCA
TGF-β3	AAGYGGGYCCATGAACCTAA	GCTACATTTACAAGACTTCAC
IL-1β	AAAAGCTTGGTGATGTCTGG	TTTCAACACGCAGGACAGG
IL-6	GACTGGAGATGTCTGAGGCTCAT	CCCAGGGAGAAGGCAACTG
IL-8	ATGACTTCCAAGCTGGGCCGTG	TATGAATTCTCAGCCCTCTTCAAAA
VEGF	GAGGCCTTGCCTTGCTGCTCTA	CACCAGGGTCTCGATTGGAT
MMP-2	AGATCTTCTTCTTCAAGGACCGGTT	GGCTGGTCAGTGGCTTGGGGTA
MMP-9	ATTTCTGCCAGGACCGCTTCTACT	CAGTTTGTATCCGGCAAACTGGCT
MMP-13	TCCCAGGAATTGGTGATAAAGTAGA	CTGGCATGACGCGAACAATA
[internal control]		
GAPDH	CCCATGTTCGTCATGGGTGT	TGGTCATGAGTCCTTCCACGATA

## Data Availability

Not applicable.

## Supplementary Materials

The following are available online at https://www.mdpi.com/article/10.3390/ph14040301/s1, Figure S1: Schematic view of the creep meter measuring method for the assessment of adhesiveness of semisolid formulation; Figure S2: In vivo effects of HA-B and -G ointments at 0.1% on healing of full-thickness excisional.
