# Supplementary figures and images for: Wound Healing Promotion by Hyaluronic Acid: Effect of Molecular Weight on Gene Expression and In Vivo Wound Closure
Source: Pharmaceuticals (Basel). 2021 Mar 28;14(4):301. doi: 10.3390/ph14040301 (PMC8065935; doi:10.3390/ph14040301)

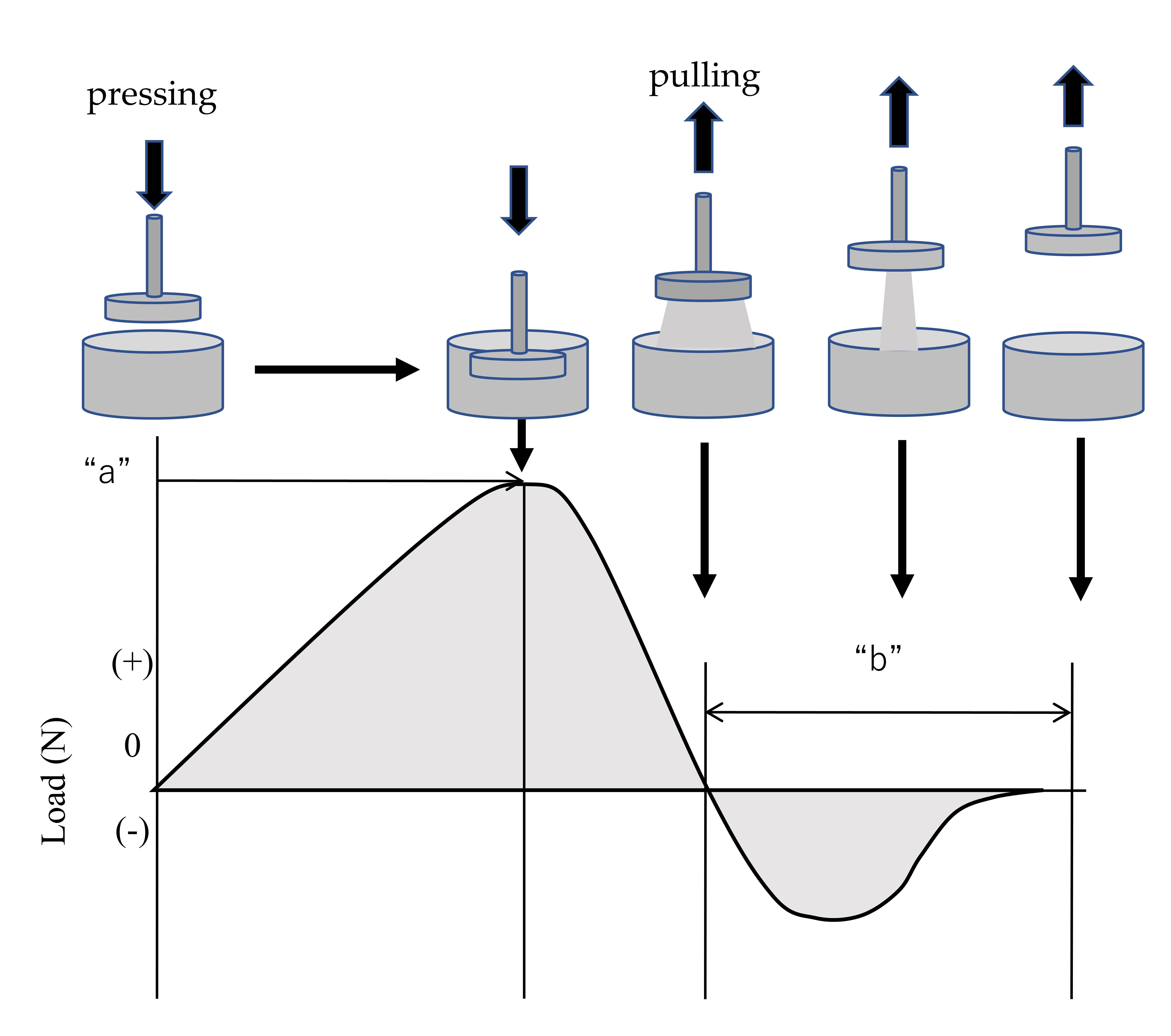

Supplement: Supplementary file 1 [file pharmaceuticals-14-00301-s001.zip › Figure S1.jpg]

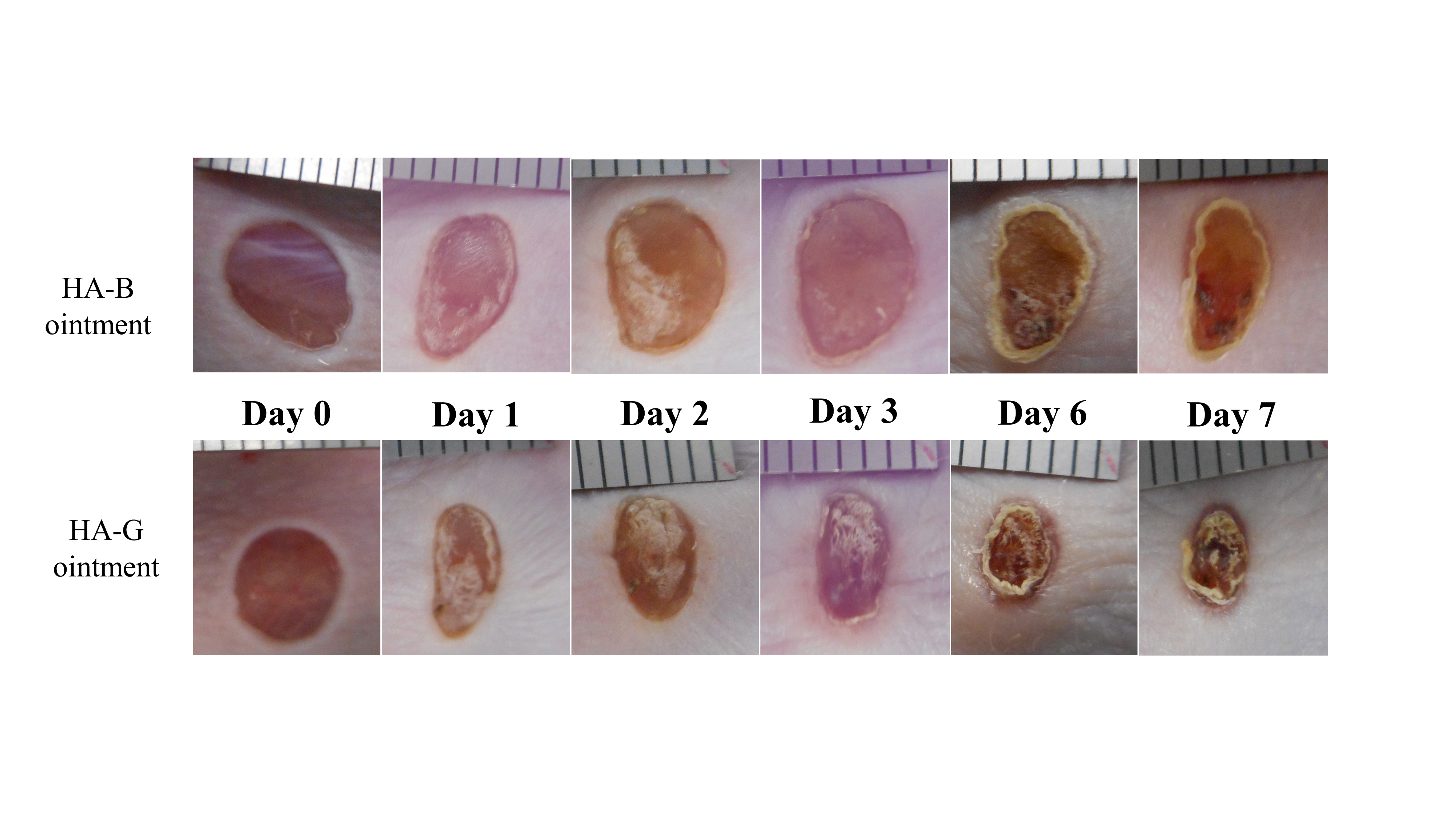

Supplement: Supplementary file 1 [file pharmaceuticals-14-00301-s001.zip › Figure S2.tif]
